# Neutralizing Activity of Broadly Neutralizing Anti-HIV-1 Antibodies against Primary African Isolates

**DOI:** 10.1128/JVI.01909-20

**Published:** 2021-02-10

**Authors:** Julio C. C. Lorenzi, Pilar Mendoza, Yehuda Z. Cohen, Lilian Nogueira, Christy Lavine, Joseph Sapiente, Marie Wiatr, Nelly R. Mugo, Andrew Mujugira, Sinead Delany, Jairam Lingappa, Connie Celum, Michael S. Seaman, Marina Caskey, Michel C. Nussenzweig

**Affiliations:** a Laboratory of Molecular Immunology, The Rockefeller University, New York, New York, USA; b Center for Virology and Vaccine Research, Beth Israel Deaconess Medical Center, Harvard Medical School, Boston, Massachusetts, USA; c Kenya Medical Research Institute, Nairobi, Kenya; d Infectious Diseases Institute, Makerere University, Kampala, Uganda; e University of the Witwatersrand, Johannesburg, South Africa; f Department of Global Health, University of Washington, Seattle, Washington, USA; g Department of Medicine, University of Washington, Seattle, Washington, USA; h Department of Pediatrics, University of Washington, Seattle, Washington, USA; i Department of Epidemiology, University of Washington, Seattle, Washington, USA; j Howard Hughes Medical Institute, The Rockefeller University, New York, New York, USA; Emory University

**Keywords:** bNAb, human immunodeficiency virus

## Abstract

Novel therapeutic and preventive strategies are needed to contain the HIV-1 epidemic. Broadly neutralizing human antibodies (bNAbs) with exceptional activity against HIV-1 are currently being tested in HIV-1 prevention trials. The selection of anti-HIV-1 bNAbs for clinical development was primarily guided by their *in vitro* neutralizing activity against HIV-1 Env-pseudotyped viruses. Here, we report on the neutralizing activity of 9 anti-HIV-1 bNAbs now in clinical development against 126 clade A, C, and D peripheral blood mononuclear cell (PBMC)-derived primary African isolates. The neutralizing potency and breadth of the bNAbs tested were significantly reduced compared to those seen with pseudotyped-virus panels. The difference in sensitivity between pseudotyped viruses and primary isolates varied from 3- to nearly 100-fold depending on the bNAb and the HIV-1 clade. Thus, the neutralizing activity of bNAbs against primary African isolates differs from their activity against pseudovirus panels. The data have significant implications for interpreting the results of ongoing HIV-1 prevention trials.

**IMPORTANCE** HIV remains a major public health problem worldwide, and new therapies and preventive strategies are necessary for controlling the epidemic. Broadly neutralizing antibodies (bNAbs) have been developed in the past decade to fill this gap. The neutralizing activity of these antibodies against diverse HIV strains has mostly been measured using Env-pseudotyped viruses, which overestimate bNAb coverage and potency. In this study, we measured the neutralizing activity of nine bNAbs against clade A, C, and D HIV isolates derived from cells of African patients living with HIV and produced in peripheral blood mononuclear cells. We found that the coverage and potency of bNAbs were often significantly lower than what was predicted by Env-pseudotyped viruses and that this decrease was related to the bNAb binding site class. These data are important for the planning and analysis of clinical trials that seek to evaluate bNAbs for the treatment and prevention of HIV infection in Africa.

## Abstract (precis)

HIV remains a major public health problem worldwide, and new therapies and preventive strategies are necessary for controlling the epidemic. Broadly neutralizing antibodies (bNAbs) have been developed in the past decade to fill this gap.

## INTRODUCTION

The development of broadly neutralizing antibodies (bNAbs) against HIV has matured in the past few years as several bNAbs were evaluated in clinical trials. VRC01 (1), 3BNC117 (2) and 10-1074 (3) have been the most extensively evaluated to date, in healthy volunteers (4, 5), in viremic people living with HIV (6–9), and in the setting of analytical treatment interruption (10–13), with encouraging results. These trials were restricted to patients living in the United States and Europe, limiting the assessment of the global utility of these antibodies, since the majority of the individuals in the regions in question were infected with clade B HIV-1 (14).

One potential limitation in the development of bNAbs is that their activity has been documented primarily using panels of Env-pseudotyped viruses. However, we (15) and others (16–19) have shown that using Env-pseudotyped viruses often overestimates both the breadth and potency of bNAbs compared to peripheral blood mononuclear cell (PBMC)-derived HIV isolates.

Here, we report on the breadth and potency of nine bNAbs currently in clinical development against primary PBMC-derived HIV-1 viruses isolated from individuals living in South Africa, Uganda, and Kenya. We compared these results with data from Env-pseudotyped virus panels as well as matched Env-pseudotyped viruses derived from the African isolates.

## RESULTS

To examine the coverage of bNAbs in clinical development against HIV-1 variants circulating in Africa, we obtained 218 cryopreserved PBMC samples from people living with HIV-1 who participated in one of three studies: the Partners in Prevention HSV/HIV Transmission Study (20), the Couples Observational Study (21), or the Partners PrEP Study (22). The samples were collected from participants recruited at sites in South Africa (*n* = 84), Uganda (*n* = 68), and Kenya (*n* = 66). Bulk CD4^+^ T lymphocytes were cultured, yielding 126 (58%) HIV-1 isolates after 21 days (Table 1).

**TABLE 1:**
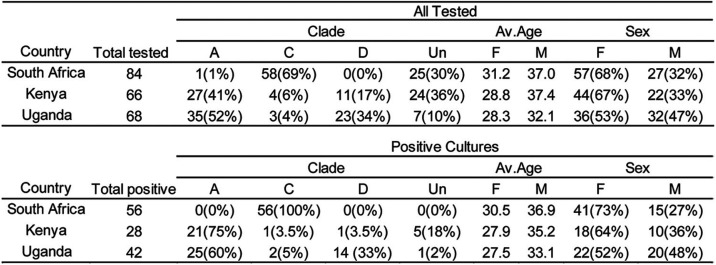
Demographical characteristics of all tested individuals^*a*^

a F, female; M, male; Un, undetermined.

**FIG 1:**
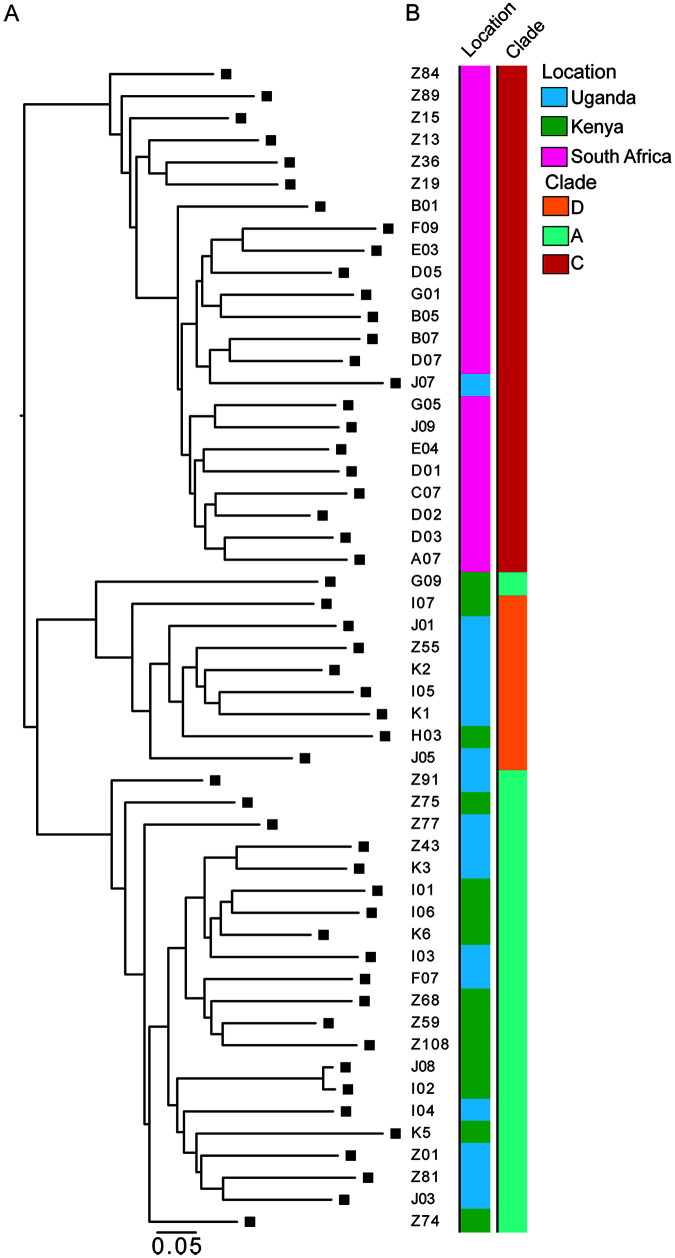
(A) Maximum-likelihood phylogenetic tree of *env* sequences of viruses isolated from outgrowth cultures by SGA. (B) The first bar to the right of the phylogenetic tree represents the country of origin of the sample; the second bar represents the HIV-1 clade of every sample.

To examine the genetic diversity of the HIV-1 viruses obtained from the cultures, we performed single-genome amplification (SGA) on 53 viral supernatants and obtained 172 independent sequences representing clades A, C, and D, with 2 sequences per supernatant on average. We observed that the viruses were phylogenetically grouped in large part by their geographic origins and clades (Fig. 1A and B).

The following bNAbs were tested for neutralizing activity against the PBMC isolates in TZM-bl assays: 3BNC117-LS, VRC01, VRC07-523LS, and 1-18, all of which are CD4 binding site specific (CD4bs), (1, 2, 23, 24); 10-1074-LS, and BG18, which target the base of the V3 glycan and surrounding glycans (3, 25); and PGDM1400 and CAP256-VRC25.26, which are specific for the V2 loop (26, 27). We also tested the combination of 3BNC117-LS and 10-1074-LS, which is currently in clinical development (9, 12) (clinicaltrials.gov/ct2/show/NCT03254277, clinicaltrials.gov/ct2/show/NCT03554408, clinicaltrials.gov/ct2/show/NCT04250636, and clinicaltrials.gov/ct2/show/NCT04173819).

**FIG 2:**
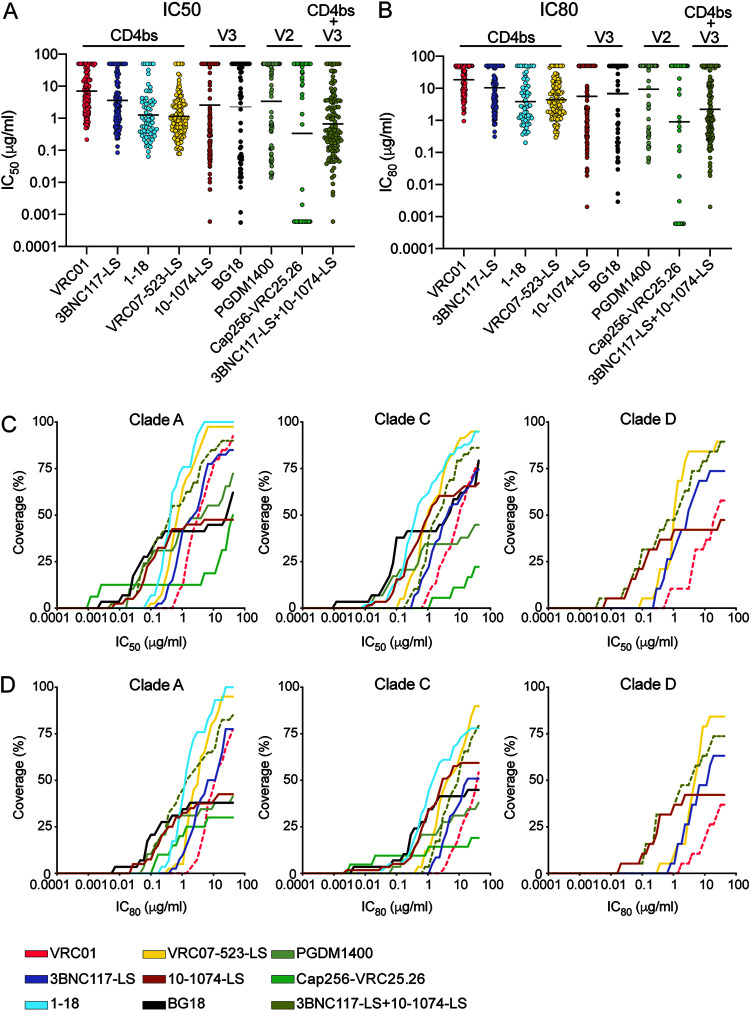
(A) Dot plot showing IC_50_s of unique PBMC-derived viruses for each bNAb tested. (B) Dot plot showing IC_80_s of unique PBMC-derived viruses for each bNAb tested. Each dot represents a single virus. Black bars represent geometric mean IC_50_s and IC_80_s. (C and D) Coverage curves. For each antibody, the graph shows the percentage of viruses neutralized in the TZM-bl assay at a given IC_50_ (C) or IC_80_ (D) for the PBMC-derived primary isolates for HIV-1 clades A, C, and D. All bNAbs are represented with the same color scheme as in panel A. The pink dotted line represents VRC01, and the olive green represents the combination of 3BNC117-LS and 10-1074-LS.

The geometric mean 50% inhibitory concentration (IC_50_) for VRC01, which is now being tested in two large efficacy prevention trials (clinicaltrials.gov/ct2/show/NCT02568215 and clinicaltrials.gov/ct2/show/NCT02716675), was 7.01 µg/ml for all viral isolates (Fig. 2A; Table 2). Only 57% of the viruses tested were sensitive to VRC01 at concentrations below 10 µg/ml (Fig. 2A to D; Table 2; also, see Data Set S1 in the supplemental material). Other CD4bs antibodies were substantially more potent than VRC01, including VRC07-523LS and 1-18, with geometric mean IC_50_s of 1.14 µg/ml and 1.27 µg/ml, respectively. These two antibodies alone covered 92% and 87% of the viruses tested at concentrations below 10 µg/ml (Fig. 2A to D; Table 2; Data Set S1). 10-1074-LS and BG18, which target the base of the V3 loop, demonstrated geometric mean IC_50_s of 2.55 µg/ml and 2.64 µg/ml, respectively (Fig. 2A to D; Table 2; Data Set S1). PGDM1400 and CAP256-VRC25.26, which target the V2 loop, demonstrated geometric mean IC_50_s of 3.38 µg/ml and 0.16 µg/ml, respectively. However, 10-1074-LS, BG18, PGDM1400, and CAP256-VRC25.26 covered only 52%, 49%, 46%, and 47% of the viruses, respectively, at concentrations below 10 µg/ml (Fig. 2A to D; Table 2; Data Set S1). The combination of 3BNC117-LS and 10-1074-LS performed better than any single antibody alone in terms of potency. The geometric mean IC_50_ for the combination was 0.65 µg/ml, and 84% of the viruses were sensitive at concentrations below 10 µg/ml (Fig. 2A to D; Table 2; Data Set S1).

**TABLE 2:**
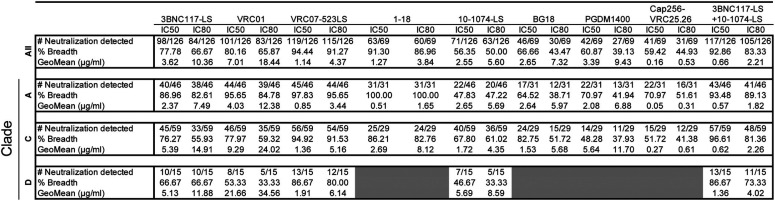
Breadth, IC_50_s, and IC_80_s in TZM-bl cells for PBMC-derived isolates

**FIG 3:**
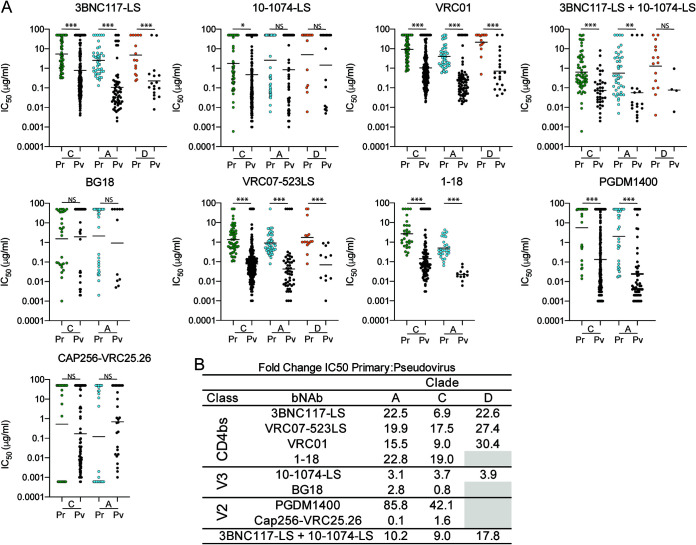
(A) IC_50_s of unique PBMC-derived viruses (Pr) shown in color, and corresponding clade pseudovirus panel in black (Pv). The viruses are organized by HIV-1 clade. Each dot represents a single virus. Black bars represent geometric mean IC_50_s. (B) Fold change between the geometric mean IC_50_s (in micrograms per milliliter) of PBMC-derived viruses and the corresponding pseudovirus panel. Statistical analysis was done using the Mann-Whitney test. Statistical significance was defined as a *P* value of <0.05 unless stated otherwise. *P* values smaller than 0.05 were considered statistically significant. *, *P* < 0.05; **, *P* < 0.01; ***, *P* < 0.001; NS, not significant.

To determine whether the sensitivity of the primary African isolates to bNAbs differs from that of standard pseudovirus panels, we compared the data obtained from the outgrowth cultures with those from well-characterized clade A, C, and D pseudoviruses (Fig. 3A and B; Tables 3 and 4). All of the bNAbs tested were more potent and showed increased breadth against the pseudoviruses compared to the primary isolates. The difference between pseudovirus and primary isolates varied between antibodies. For example, CD4 binding site-specific bNAbs showed significant average decreases in potency of 20-, 13-, and 27-fold for primary isolates from clades A, C, and D, respectively (*P < *0.0001 for all clades tested for CD4 binding site-specific antibodies). Moreover, these antibodies neutralized an average of 4.2%, 13.5%, and 28.1% fewer clade A, C, and D primary isolates, respectively, when tested against primary isolates than against pseudoviruses at concentrations below 10 µg/ml.

**TABLE 3:**
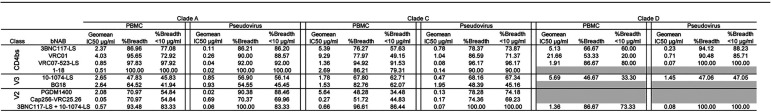
Breadth, IC_50_s, and IC_80_s in TZM-bl cells for PBMC-derived isolates and pseudovirus panels

**TABLE 4:**
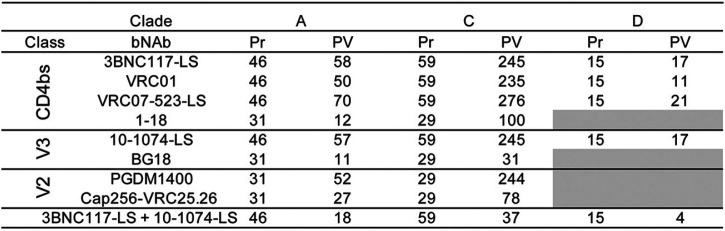
Number of tested samples for PBMC derived isolates (Pr) and pseudovirus panels (PV)

The difference in potency and breadth between pseudoviruses and primary isolates was less dramatic for bNAbs targeting the V3 glycan. On average, there was only a 3-fold difference in IC_50_ between primary isolates and pseudovirus panels for clades A, C, and D (*P = *0.002 for 10-1074-LS on clade C; the difference was not significant for clades A and D for both 10-1074-LS and BG18). V3 glycan antibodies also retained most of their breadth, as shown by the numbers of strains reaching IC_50_s at concentrations below 10 µg/ml (Fig. 3A and B; Tables 3 and 4).

The two V2-loop bNAbs were unusual in that they had very different relative potencies against primary and pseudotyped clade A and C viruses. Whereas CAP256-VRC25.26 showed no significant difference in activity, PGDM1400 was 85- and 42-fold less active against primary clade A and C viruses than pseudotyped viruses, respectively (*P < *0.0001) (Fig. 3A and B; Tables 3 and 4). These antibodies neutralized 24% and 32% fewer clade A and C primary isolates, respectively, than pseudoviruses at concentrations below 10 µg/ml. Finally, the 3BNC117-LS/10-1074-LS combination was on average 12-fold less active against the primary isolates than pseudoviruses and showed no decrease in breadth for clade A but did show 13.5% and 26.5% decreases in breadth with regard to the numbers of strains reaching IC_50_s at concentrations below 10 µg/ml for clades C and D, respectively (Fig. 3A and B; Tables 3 and 4).

**FIG 4:**
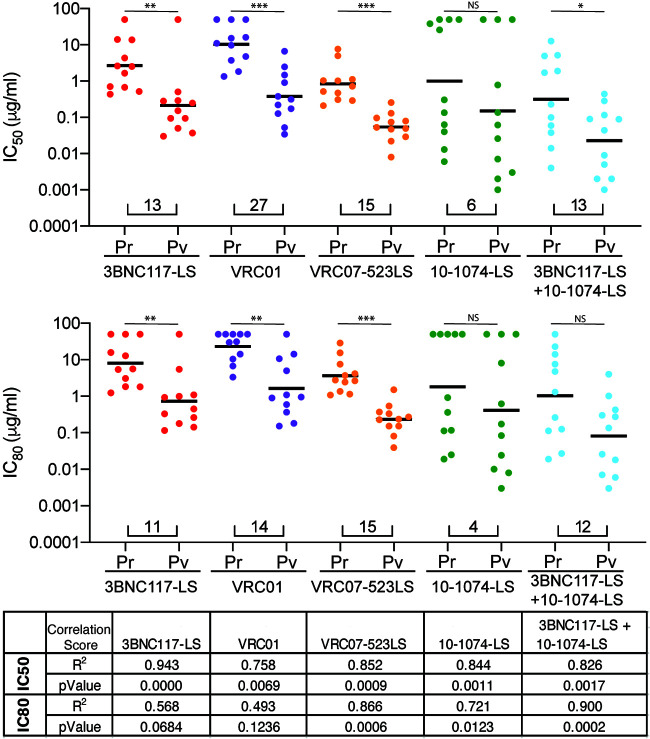
(Top and middle) IC_50_s and IC_80_s of unique PBMC-derived clonal viruses (Pr) and corresponding pseudoviruses (Pv) for each antibody. Each dot represents a single virus. Black bars represent geometric mean IC_50_s. Numbers under the dots indicate the fold change in geometric mean IC_50_s between the 2 groups. Statistical analysis was done using the Mann-Whitney test. Statistical significance was defined as a *P* value of <0.05 unless stated otherwise. *P* values smaller than 0.05 were considered statistically significant. *, *P* < 0.05; **, *P* < 0.01; ***, *P* < 0.001; NS, not significant. (Bottom) Correlation analysis between Pr and Pv. *r*^2^ and *P* values were obtained from the Pearson correlation coefficient.

To determine whether the differences between primary isolates and pseudovirus panels were attributable to sequence differences between the viruses being tested, we cloned HIV-1 *env* genes from 11 different primary cultures, expressed them as pseudotyped viruses, and tested them against a panel of 5 bNAbs in the TZM-bl neutralization assay. IC_50_s and IC_80_s for the PBMC-derived viruses and the matched pseudoviruses showed similar fold differences than those found between primary isolates and pseudovirus panels for all bNAbs tested (Fig. 4). Besides the underestimation of the resistance levels of to bNAbs presented in the pseudovirus experiments, we observed a significant correlation between the results of both experiments (Fig. 4). The data suggest that there are significant differences in bNAb potency and breadth between primary clade A, C, and D isolates and pseudotyped viruses and that the magnitude of these differences is bNAb specific.

## DISCUSSION

We measured the neutralization profile of nine bNAbs currently in clinical development on 126 primary isolates obtained from PBMC cultures from individuals infected with HIV-1 clades A, C, and D. VRC01, the most advanced clinical candidate, is nearly 15 times less active against primary isolates than pseudotyped viruses. Similar results were obtained with other CD4 binding site antibodies. In contrast, the two V2-directed antibodies tested varied widely in their ability to neutralize pseudotyped viruses and primary isolates. Thus, the results obtained with pseudotyped virus panels cannot be translated directly to bNAb activity on primary isolates.

Our results extend earlier work with less potent antibodies (16–18) and with bNAbs against clade B viruses (15) to clades A, C, and D. In all cases, primary isolates were less sensitive to bNAbs than pseudotyped viruses. However, the relative reduction in activity differed between antibodies that target different epitopes on the envelope spike, with V3 glycan bNAbs 10-1074-LS and BG18 being least affected and PGDM1400 the most affected. In addition, the magnitude of the differences varies among viral clades. Combinations of bNAbs, as exemplified by 3BNC117-LS and 10-1074-LS, are advantageous in this respect, as also suggested by *in vitro* and *in silico* analysis using Env- pseudotyped panels (28).

A number of non-mutually exclusive hypotheses have been suggested to explain the enhanced susceptibility of 293T-derived pseudotyped viruses to neutralization by bNAbs. For example, sensitivity to neutralization could be dependent on the number of envelope protein spikes, with fewer spikes bound on the surface of 293T-derived pseudotyped viruses than PBMC-derived primary isolates (16, 18). Another possibility involves differential glycosylation by different packaging cell types. bNAbs frequently target glycan-dependent epitopes; therefore, the differential glycosylation profile of the envelope spike produced in different cell types could also alter their neutralization profile (49, 50). However, V3 glycan bNAbs and CAP256-VRC25.26, which target highly glycan-dependent epitopes, were the least affected. Similarly, PG9, a V2 peptide glycan-specific bNAb (29, 30), showed only small changes in its neutralization profile between clade B pseudotyped viruses and PBMC-derived viruses (18). Still another possibility is that most of the pseudoviruses tested in the standard panels were isolated between 1998 and 2010, whereas our samples were collected between 2007 and 2012 (31), and there appears to be increased bNAb resistance over time (32–35).

Clinical trials testing bNAbs for HIV-1 prevention are now being conducted in Africa and other parts of the world. The largest of these trials is testing VRC01 at several sites in Africa (Botswana, Kenya, Malawi, Mozambique, South Africa, Tanzania, and Zimbabwe), where the majority of the HIV-1 infections are caused by clade A, C, and D viruses (14). Although the results of those trials are not yet known, data are available from smaller trials where bNAbs were administered to individuals undergoing analytical treatment interruption (ATI). In the absence of antiretroviral therapy, nearly all participants experience viral rebound in 2 to 3 weeks, and it is believed that recrudescence of viremia is due to reactivation of HIV-1 from latently infected CD4^+^ T cells (36). Single antibodies were able to decrease viremia levels or delay the return of quantifiable viremia, but their ability to do so correlated with their neutralizing activity against primary isolates and not pseudotyped viruses (6, 7, 10, 11, 37, 38). For example, VRC01 had little measurable effect on delaying HIV-1 rebound when administered during ATI (13, 39). In contrast, antibody combinations maintain suppression of viremia during ATI in individuals harboring bNAb-sensitive viruses for as long as antibody concentrations remain above 10 µg/ml (12). Should the clinical outcomes in the ongoing VRC01 prevention trials track with bNAb activity against primary isolates as opposed to pseudotyped virus panels, there could be up to a 15-fold difference between the predicted and observed outcomes of the trial. Nevertheless, by analogy with the ATI trials, if the AMP trials demonstrate even a smaller-than-projected effect with VRC01, it provides a proof of concept that passive immunization can prevent sexual transmission of sensitive HIV-1 strains and indicates that combinations should be highly effective.

## MATERIALS AND METHODS

### Samples.

The study was conducted with the approval of The Rockefeller University Institutional Review Board. Samples were collected during the course of three studies in sub-Saharan Africa. (i) The first is the Partners in Prevention HSV/HIV Transmission Study. Between November 2004 and April 2007, 3,408 HIV-serodiscordant heterosexual couples were enrolled from 14 study sites in sub-Saharan Africa into this phase III clinical trial evaluating the efficacy of herpes simplex virus 2 (HSV-2) suppressive therapy (acyclovir 400 mg orally twice daily versus matching placebo) provided to persons infected with both HIV-1 and HSV-2 who had CD4 counts of ≥250 at enrollment to prevent HIV transmission to their HIV-uninfected heterosexual partner (20). (ii) The second was the Couples Observational Study. A total of 485 HIV-serodiscordant heterosexual couples were recruited at two of the same sites as the Partners in Prevention HSV-2/HIV Transmission Study (Kampala, Uganda, and Soweto, South Africa) for a prospective, observational study of biologic correlates of HIV protection; there was no HSV-2 coinfection or CD4 count enrollment requirement (21). (iii) The third was the Partners PrEP Study. This was a randomized, phase III clinical trial of antiretroviral pre-exposure chemoprophylaxis (300 mg tenofovir once daily versus 300 mg tenofovir plus 200 mg emtricitabine once daily versus matching placebo) conducted at nine sites in Kenya and Uganda (22).

### CD4^+^ T cell outgrowth culture.

Bulk outgrowth cultures were performed as previously described (11). Briefly, PBMCs were obtained from HIV-1-infected individuals, and CD4^+^ T lymphocytes were isolated by negative selection with magnetic beads (Miltenyi). A total of 2 × 10^6^ CD4^+^ T lymphocytes were activated using anti-CD3/CD2/CD28 beads (Miltenyi) and cultured in the presence of 100 U/ml interleukin 2 (IL-2) (Peprotech) at 37°C and 5% CO_2_. CD4^+^ T lymphocytes were cocultured with irradiated heterologous PBMCs from healthy donors (1 × 10^6^). After 24 h of activation, 1 × 10^5^ Molt 4 CCR5 cells were added. The medium was replaced twice a week, and the presence of p24 in the culture supernatant was quantified by the Lenti-X p24 Rapid Titer kit (Clontech) after 7, 14, and 21 days of culture. The infectivity of viral cultures was confirmed by a 50% tissue culture infective dose assay with TZM-bl cells (40). We performed a single outgrowth culture for each tested individual (*n* = 218) and further analyzed the ones with a positive enzyme-linked immunosorbent assay (ELISA) signal (*n* = 126).

### Neutralization assays.

TZM-bl cell neutralization assays were performed as previously described (40, 41). Neutralization assays were conducted in laboratories meeting good clinical laboratory practice quality assurance criteria. All bulk outgrowth culture primary isolates were tested against 3BNC117-LS, VRC01, 10-1074-LS, VRC07-523LS and the combination of 3BNC117-LS and 10-1074-LS. Sixty-nine were also tested against PGDM1400 (provided by Dennis Burton, Scripps Research Institute), BG18, 1-18 (provided by Florian Klein, University of Cologne), and CAP256-VRC25.36 (provided by John Mascola, NIH Vaccine Research Center). The maximum antibody concentration tested was 50 µg/ml. All the experiments were performed in triplicate. Neutralization values used in Fig. 3 for the pseudovirus panel were obtained from the Antibody Database software (42), the CATNAP database (43), or the original reports for antibodies 1-18 (25), BG18 (24), and the combination of 3BNC117-LS and 10-1074-LS (44).

### Virus sequence analysis.

HIV *env* sequences from p24-positive supernatants were obtained and analyzed as previously described (45). Sequences derived from each bulk culture that had double peaks (cutoff consensus identity for any residue, <75%) or stop codons or were shorter than the expected envelope size were omitted from downstream analysis. Phylogenetic analysis was performed by generating nucleotide alignments using MAFFT (46) and posterior phylogenetic trees using PhyML v3.1 (47), using the GTR model with 1,000 bootstraps. Clade determination was performed using the NCBI subtyping tool (http://www.ncbi.nlm.nih.gov/projects/genotyping/formpage.cgi). For samples not sequenced in this study, the clade was determined by sequencing a 514-bp region of the *env* gene (C2-V3-C3 region) from plasma samples as previously described (20–22).

### Pseudotyped-virus production.

Pseudotyped-virus production was performed as previously described (48). The cytomegalovirus (CMV) promoter was amplified by PCR from the pcDNA 3.1D/V5-His-TOPO plasmid (Life Technologies) with forward primer 5′-AGTAATCAATTACGGGGTCATTAGTTCAT-3′ and reverse primer 5′-CATAGGAGATGCCTAAGCCGGTGGAGCTCTGCTTATATAGACCTC-3′. A 1-µl volume of the first-round PCR product from each individual *env* gene obtained from bulk cultures was amplified with primers 5′-CACCGGCTTAGGCATCTCCTATGGCAGGAAGAA-3′ and 5′-ACTTTTTGACCACTTGCCACCCAT-3′. PCR products were purified with the Macherey-Nagel gel and PCR purification kit. The CMV promoter amplicon was fused to individual *env* genes by overlap PCR with 10 ng of *env* and 0.5 ng of CMV with forward primer 5′-AGTAATCAATTACGGGGTCATTAGTTCAT-3′ and reverse primer 5′-ACTTTTTGACCACTTGCCACCCAT-3′. Resulting amplicons were analyzed by gel electrophoresis, purified with the Macherey-Nagel gel and PCR purification kit, and cotransfected with pSG3Δenv backbone vector (NIH AIDS Reagent Program) into HEK293T cells to produce pseudoviruses as previously described (48).

### Statistical analysis.

Statistical analyses were performed with GraphPad Prism 8.0 software. Statistical analysis presented in Fig. 3 and 4 were analyzed using the Mann-Whitney test. Correlations were tested by Pearson correlation coefficient. Statistical significance was defined as a *P* value of <0.05 unless stated otherwise. *P* values smaller than 0.05 were considered statistically significant. The data are shown as means and individual data points.

## Supplementary Material

Supplemental file 1

Supplemental file 2
